# Prey selection along a predators’ body size gradient evidences the role of different trait-based mechanisms in food web organization

**DOI:** 10.1371/journal.pone.0292374

**Published:** 2023-10-05

**Authors:** Esteban Ortiz, Rodrigo Ramos-Jiliberto, Matías Arim

**Affiliations:** 1 Departamento de Ecología y Gestión Ambiental-Centro Universitario Regional del Este, Universidad de la República, Maldonado, Uruguay; 2 GEMA Center for Genomics, Ecology & Environment, Universidad Mayor, Santiago, Chile; Swedish University of Agricultural Sciences and Swedish Institute for the Marine Environment, University of Gothenburg, SWEDEN

## Abstract

An increase in prey richness, prey size and predator trophic position with predator body size has been consistently reported as prime features of food web organization. These trends have been explained by non-exclusive mechanisms. First, the increase in energy demand with body size determines that larger predators must reduce prey selectivity for achieving the required number of resources, being consumption relationships independent of prey traits. Second, when consumption is restricted by gape limitation, small predators are constrained to select among small prey. However, this selection weakens over large predators, which progressively consume more and larger prey. Finally, the optimal foraging mechanism predicts that larger predators optimize their diet by selecting only large prey with high energy reward. Each one of these mechanisms can individually explain the increase in prey richness, prey size and predator trophic position with predator body size but their relative importance or the direct evidence for their combined role was seldom considered. Here we use the community assembly by trait selection (CATS) theory for evaluating the support for each one of these mechanisms based on the prey selection patterns that they predict. We analyzed how prey body size and trophic guild determine prey selection by predators of increasing body size in a killifish guild from a temporary pond system. Results support the combination of the three mechanisms to explain the structural trends in our food web, although their strength is contingent on prey trophic group. Overall, high energy prey are preferred by larger predators, and small predators select small prey of all trophic status. However, large predators prefer large primary producers and avoid large carnivorous prey, probably because of the inherent risk of consuming other carnivorous. Our study provides a mechanistic understanding of how predator traits determine the selection of prey traits affecting food web assembly.

## Introduction

**Fig 1:**
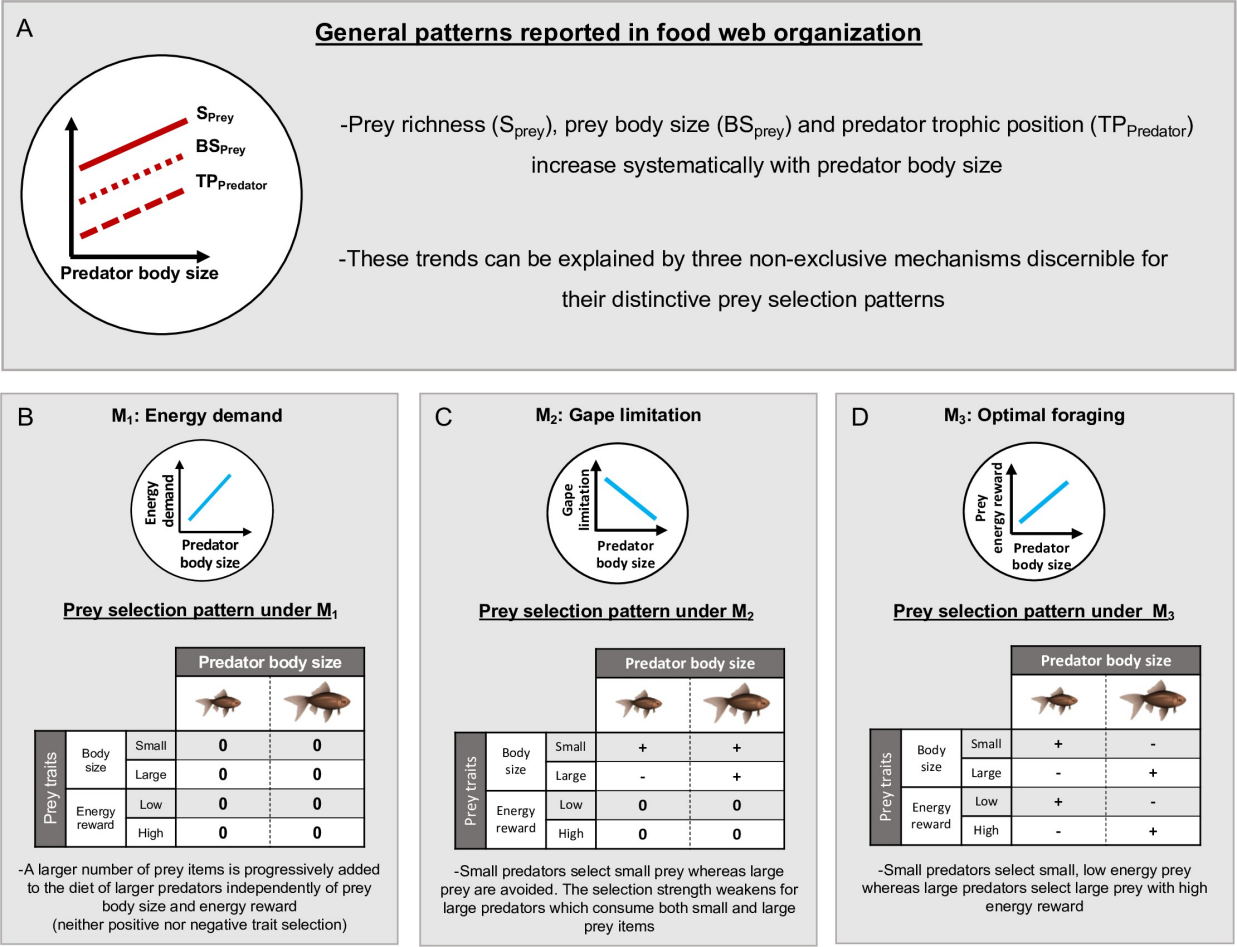
Mechanisms and hypotheses. (A) Common structural patters broadly reported in food webs. Along a gradient in predator body size, an increase in prey richness, prey body size and predator trophic position have been systematically observed in numerous food webs. These trends can be explained by three non-exclusive mechanisms discernible for their distinctive prey selection patterns based on predator body size and prey traits (i.e. size and energy reward). (B-D) Mechanisms proposed to explain the structural trends shown in (A). (B) Under the energy demand mechanism (M_1_), the increase in prey consumption by large predators is determined by the positive association between predator´s energy demand and body size. The progressive incorporation of prey items into the diet of larger predators is not associated with prey traits, involving a trait-independent consumption pattern. (C) The gape limitation mechanism (M_2_) allows large predators to consume over more and larger prey items due to the relaxation of gape limitation. This mechanism determines a selection pattern in which small predators select only small prey, with large prey being excluded from the diet. This preference for small prey weakens as predator body size increases, since large predators are able to consume all size classes of prey. (D) Under the optimal foraging mechanism (M_3_), prey items are consumed according to their energy reward. This mechanism determines that larger predators select larger prey with higher energy reward.

Species interactions are highly influenced by species body size [1–3]. Predator-prey body size relationships determine variations in community-level properties such as the distribution of interaction strength [4, 5], secondary extinctions after species loss [6], and community stability [7]. Consequently, empirical studies have reported robust trends in trophic interaction properties with increasing body size of predators (Fig 1A). Of most importance, the increases in prey richness and in prey size were widely reported at the intra and interspecific level [1, 3, 8–15], and have been identified as important determinants of food web structure and stability [2, 3, 11, 12, 16–18]. Therefore, body size, through determining predator-prey interactions, potentially shapes community level structural and functional attributes beyond pairwise ecological relationships.

Prey selection is a leading process modulating predator-prey relationships [19]. Specific mechanisms have been proposed to explain observed trends in prey selection as a function of the body size of predators and their prey [12, 20]. These mechanisms include: first, the increase in the number of prey items consumed due to the need to satisfy the increase in energy demand at larger body sizes [21, 22] (Fig 1B); second, the progressive relaxation of gape limitation, which allows the gradual consumption over more and larger prey [23, 24] (Fig 1C); and third, the optimal foraging mechanism, which predicts strengthening the preference for prey of larger size–with greater energy reward–as predator become larger [25–27] (Fig 1D). These mechanisms determine specific prey selection patterns governed by the interaction between prey traits and predator body size [12, 20], and may operate either individually or through their combined action. The increase in energy demand with predator body size determines that larger predators progressively incorporate prey items into their diet independently of prey traits—e.g. predators become less selective and more generalists [12]. This mechanism does not involve changes in the selection of prey with different traits and represents a null hypothesis from the perspective of prey traits (Fig 1B). However, gape limitation is expected to mediate the association between predator and prey body sizes [1, 8, 12, 23, 28]. The mechanism of gape limitation operates through excluding large prey from being consumed by small predators, determining a negative selection on these items and a preference over small prey. This selection pattern progressively weakens as predators become larger being both small and large prey equally preferred by large predators (Fig 1C). Finally, since the energetic content of a prey is positively associated with its trophic level and body size [21, 29, 30], the higher energy demand of large predators may consequently determine a selection for larger-bodied prey that provide greater energetic return (e.g. animals over basal resources) (Fig 1D) [25–27]. These three prey selection models evidence the basic mechanisms underlying body size dependent trends in predator trophic position [24], prey richness [8, 17], and prey size [28] (Fig 1A), and indirectly, emergent structural community properties such as nestedness [13, 14] and modularity [27]. Remarkably, although these mechanisms generate explicit predictions about the body size dependent prey selection behavior, they have often been indirectly evaluated. This has been done mainly through interpretating patterns of over–versus under–representation of prey traits relative to the environmental offer, along a gradient of predator body size (e.g. [31–33]).

New methods for analyzing trait selection along ecological gradients have been extensively developed. Among them, the community assembly through trait selection (CATS) theory [34–36] introduced techniques for directly evaluate the role of traits as determinants of the relative abundance of species along environmental gradients. Essentially, by means of generalized linear models, species abundances are related to species traits, environmental conditions, and the interaction among them [35–37]. This method is also suited for the analysis of trends in trophic interactions with predator body size mediated by prey traits. In this context, predator body size represents the environmental gradient and prey traits determine a selection process in consumption that could systematically change along the gradient of predator body size. Additionally, the identity of the observed prey items in the diet represents the complete prey pool consumed by all predators in the defined system. This approach overcomes the classic limitation of identifying the total available prey set, needed to infer selection mechanisms through traditional methods [25, 38, 39].

In using the CATS framework, we advance our understanding of trophic relationships by directly evaluating the support for the three alternative mechanisms that could explain the patterns of prey consumption as a function of predator body size and prey traits. The combination of the three alternative mechanisms of prey selection gives rise to seven competing hypotheses that guide our study (Fig 1D). H_1_: the increase in energy demand by larger predators determines the increase in prey abundance and the consequent diversification of diet items independently of prey traits. H_2_: consumption is determined by restrictions imposed by consumers body size (i.e. gape limitation). The banned consumption of large prey determines a negative selection for these items and/or a positive selection for smaller ones by small predators, and the progressive attenuation of this selection regimen along larger consumers. H_3_: consumption is determined by a maximization of energy acquisition. Large predators positively select big prey with high energy reward (e.g. animal prey) and avoid the consumption of small prey with low energy reward (e.g. primary producers). H_4_: relaxation of gape limitation and the increase in energy demand jointly determine the trend in diet with body size. H_5_: the increase in energy demand and the maximization of energy reward guide consumers diet, increasing selection for large prey along large consumers. H_6_: the relaxation of gape limitation and the maximization of energy reward determine that large predators select over large prey with high energy reward and small predators select small prey. H_7_: in addition to gape limitation and maximization of energy reward, the increase in energy demand with predator body size also explains the change in trophic interactions by an increase in consumption. We test the above hypotheses by analyzing our own dietary trait data from a guild of annual killifish species. This empirical system has been thoroughly studied and employed as a research model to reveal how trait-based trophic interactions affect food web structure [12, 14, 40, 41].

## Materials and methods

### Ethic statement

We used the data collected and published in [12, 42], and no additional animals were sampled or euthanized for the current study. Fish sampling in [42] was carried out in strict accordance with the recommendations of “Comisión Honoraria de Experimentación Animal (CHEA)” in 2006. Fish individuals were sampled with a hand net, euthanized with an overdose of 2-phenoxyethanol, and fixed with 4% formaldehyde. All efforts were made to minimize suffering. All specimens analyzed were placed in the Fish Collection of Facultad de Ciencias (Faculty of Science), Montevideo, Uruguay, under the Institutional Code: ZVC-P. Fishes were collected on a private land in the region of Humedales del Este (Eastern Wetlands) which was declared Biosphere Reserve by UNESCO (program MAB). Permissions to collect had been granted by the landowners.

### Study system

Predators and prey for analysis come from a set of temporary ponds located in grassland in the Laguna de Castillos basin, Rocha, Uruguay. Ponds are formed from ground depressions during the rainy season, when rainwater supply surpasses water loss due to evaporation [42]. The aquatic system shows a high diversity of macrophyte, macroinvertebrate and vertebrate species [40, 43]. Our analyses are based on the diet information collected from stomachs of 619 individuals belonging to the four killifish species that inhabit the ponds: *Austrolebias viarius*, *A*. *cheradophilus*, *A*. *lutheoflammulatus* and *Cynopoecilus melanotaenia* [12, 43]. In our study system, these species are the top predators, showing a strong positive intraguild relationship between their body mass and their trophic position, along with prey richness and number of energy sources (i.e. plants, detritus, phytoplankton and terrestrial prey) [12]. In addition, we detected a smooth incorporation of prey items with increasing body size, determining the emergence of a strong nested size-dependent organization of trophic interactions [14]. Fish individuals were sampled during winter 2006, identified at the species level and their standard length was measured following established procedures [40, 42, 44]. Following [12] and [14], individual predators were sorted by size and classified into 20 body size classes, each of them composed of 31 individuals (30 in the largest class) (S1 Fig).

Prey items in the stomach content of each individual fish were identified to the highest possible taxonomic resolution and categorized into trophic groups as primary producers, herbivorous and detritivores, and carnivorous (data published in [12, 42]). We used the membership to a trophic group as a categorical trait of prey, subsequently included in the predator selection analyses. The trophic groups represent an increasing gradient in the energy quality of prey form primary producers to carnivorous species [12, 21]. Prey size was the second trait of prey considered in the selection analyses. Prey size was estimated from a dataset of invertebrates that inhabit the pond system, that were collected in October 2008 [40] (S2 Fig). In addition, we obtained from literature [45–49] the body sizes of those items that appeared in the killifish diet but that were not collected in the field survey. These data represented less than 1% of prey items in terms of richness and abundance.

### Prey trait representation in predator body size classes

The representation of traits in communities along an environmental gradient is usually visualized through the trend exhibited by the “community-weighted mean” along the gradient [50]. This metric captures the average representation of traits in each community. In analogy, we estimated a “diet-weighted mean” (DWM), indicating the representation of prey traits across predator size classes. For quantitative traits, $DWM=\sum_{i=1}^{n}p_{i}\;*\;{\text{trait}}_{i}$, where *p*_*i*_ is the relative abundance of the trophic item *i* in a killifish size class and *trait*_*i*_ is the mean trait value of that item—that is the average prey trait value independently of its taxonomic identity. For categorical traits with *n* levels, *trait*_*i*_ represents a particular level *i* of the trait and *p*_*i*_ is the relative abundance of all prey items within *i*—that is the frequency of a categorical prey level (e.g. carnivorous prey) in predator diet, independently of prey taxonomy [50]. Here, DWM provides an estimation of the relative frequency of primary producers, herbivorous and detritivores, and carnivorous prey items, and the average prey size (combining all trophic groups) in the diet of each killifish body size class [51].

### CATS regression model and hypotheses evaluation

In the present analysis we employed CATS regression model to relate the abundance of a prey item with the interaction between its traits and the body size of consumers [34–36, 51, 52]. This technique allows to evaluate how influential the traits of a prey item are in determining its abundance in the diet of consumers with different body sizes. The CATS regression model consists of a generalized linear mixed model of Poisson family, with an offset that represents a prior abundance distribution of prey items. That is, the frequency of each prey item along all size classes of predators combined [36]. Using this approach, the abundances of prey items in each predator body size class were related to predator body size, prey traits—size and trophic group—and the interaction among them. The sign of the model coefficients (β) directly translates into positive (β > 0), null (β = 0) or negative (β < 0) prey selection according to their traits [36]. In addition, the numeric value of the model coefficients represents a measure of the relative selection for some kind of prey over others (e.g. small vs large prey). That is, the larger the magnitude of the coefficient, the larger the selection strength for a certain prey category [35, 37]. This allowed us to analyze whether and to what extent predators with different body sizes prefer different prey items (e.g. vegetable vs. animal prey) and whether this selection is affected by prey traits (e.g. body size and trophic category).

We used the CATS regression model to evaluate the prey selection patterns expected under the three alternative mechanisms, and then the support for the seven competing hypotheses (Fig 1). The inclusion of the predators and prey traits as fixed effects in the model represents a direct way of testing the predictions associated with mechanisms M_2_ and M_3_ (Fig 1B and 1C). Under the gape limitation mechanism M_2_, small predators are expected to select small prey for all trophic categories (i.e. β_small prey_ > β_large prey_), whereas this preference should weaken for large predators (i.e. β_small prey_ ≈ β_large prey_) (Fig 1B). Under the optimal foraging mechanism M_3_, large predators should select prey with high energy reward (i.e. large, animal prey—β_small prey_ < β_large prey_ and β_primary producers prey_ < β_animal prey_), but small predators may comparatively prefer prey items with low energy reward (i.e. small, primary producer prey—β_small prey_ > β_large prey_ and β_primary producers prey_ > β_animal prey_) (Fig 1C). The prediction associated to the energy demand mechanism M_1_ was tested by including the identity of predator body size class as random effect [53] (Fig 1A). Under this mechanism, it is expected that prey items are incorporated into the diet of large predators independently of prey traits. The random effect associated to the predator size class controls for the variation in consumption rate among body size classes of predators not related to prey traits. That is, trends in diet are explained by differences in consumption rate along predator body size classes due to the increasing energy demand of larger predators and are independent of changes in the selection of prey traits. Since the energy demand mechanism predicts that this variation in prey consumption originates from differences in predator body size, we also computed the Pearson correlation between the number of prey items and the predator body size.

The CATS regression model including both fixed and random effects (i.e. full model) was compared with alternative simpler models that could be used to test the mechanisms herein evaluated. The full model was compared against several nested models constructed by systematically removing different terms from the full model. The criteria used to select the best model was based on the Akaike´s Information Criterion (AIC) and the estimation of the weight of evidence [54], which indicates the probability that one of the models is the real one, as compared to all the alternative models. In addition, we performed the Wilks´ likelihood ratio test, which permits comparing the degree of empirical support that two nested statistical models receive from data. Finally, the identity of prey items was also included as random effect [53] to control for the effect of the natural differences in prey abundances—i.e. those not related to predator body size. All analysis were conducted in R [55] at α = 0.05 significance.

### Data availability

All raw data and code lines used in this study are freely available at https://doi.org/10.5061/dryad.bk3j9kdjg

## Results

**Fig 2:**
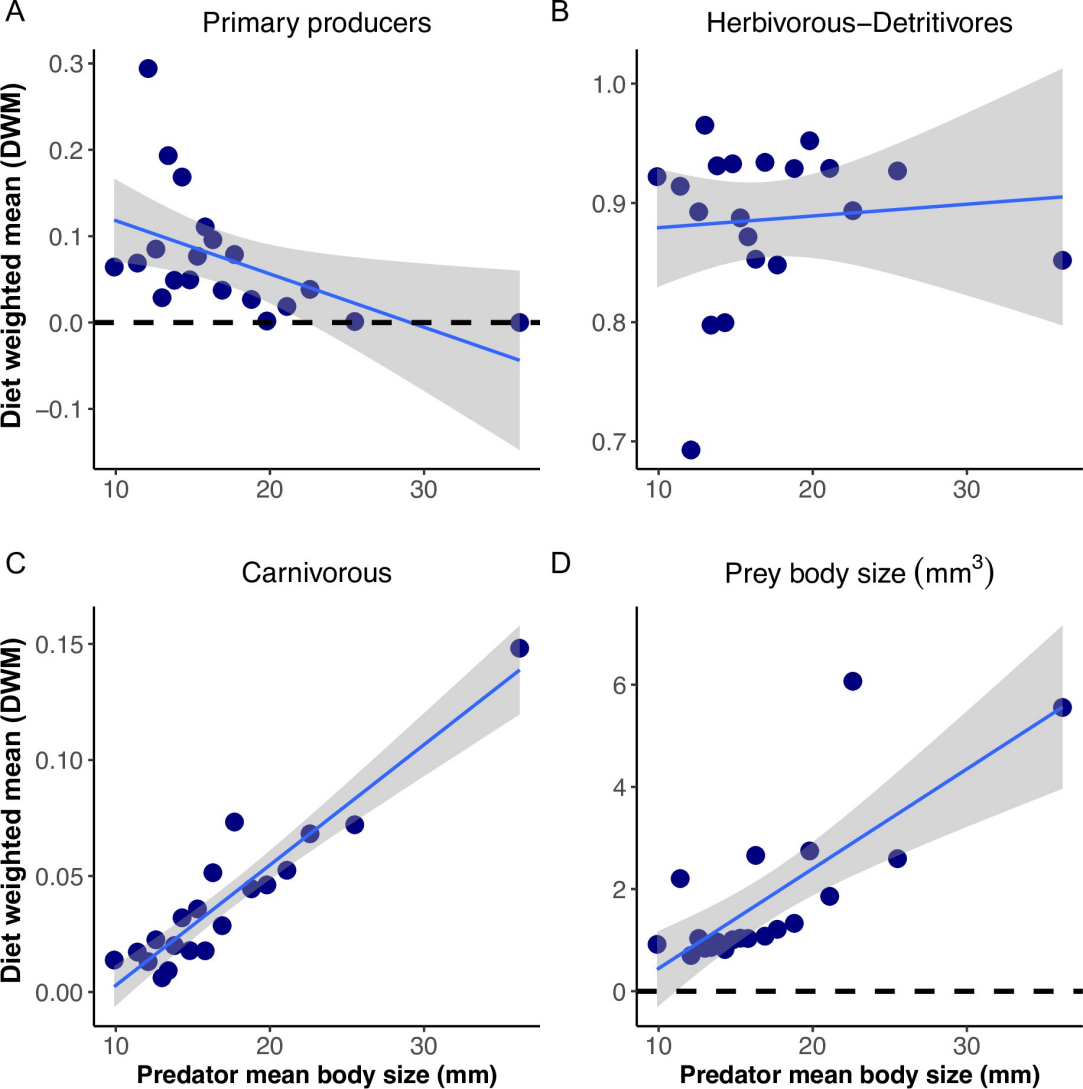
Diet weighted mean of prey traits along the gradient in predator body size. (A) primary producers are overrepresented in the diet of smaller predators, but their representation progressively decreases along size classes (R^2^ = 0.26, P = 0.02). (B) The representation of herbivorous and detritivores prey did not change along body size classes of consumers (R^2^ = 0.01, P = 0.9). (C) Carnivorous prey systematically increases with consumers body size (R^2^ = 0.88, P<0.01). (D) The average body size of prey items systematically increases with predator body size (R^2^ = 0.6, P<0.01).

We found significant trends for the diet weighted mean of prey traits along the gradient of predator body sizes. First, we observed a reduction in the proportion of primary producers present in the diet, down to a complete absence of this item in the largest predator size classes (Fig 2A). Second, the frequency of herbivorous and detritivores prey items did not differ among predators (Fig 2B). Third, we detected a strong increase in the frequency of carnivorous prey items with predator size (Figs 2C and S3). Finally, the representation of larger prey items systematically increased with predator body size (Fig 2D). It is important to note that these results only describe general patterns regarding the representation of different types of prey in the diet of predators, but do not evidence the mechanisms responsible for such patterns.

**Fig 3:**
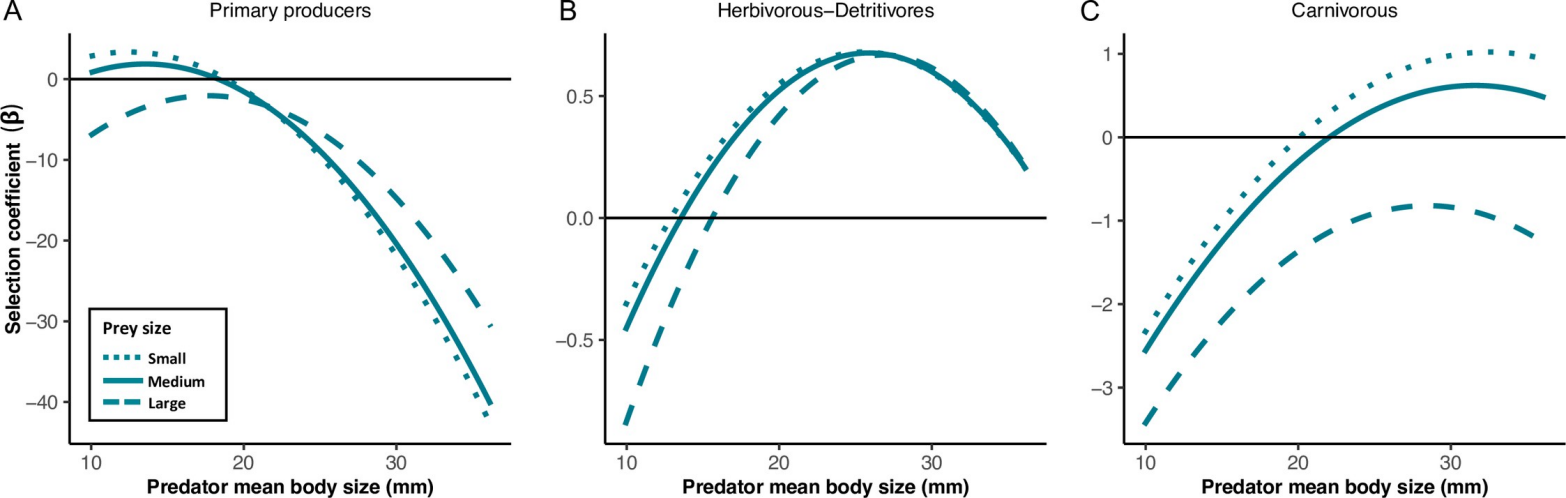
Selection of prey traits along the predator body size gradient. (A) primary producers, (B) herbivorous and detritivores and (C) carnivorous prey items. Line type shows the interaction between prey trophic status and prey body size. The dotted lines correspond to prey items with small body sizes (percentile 0.05 of prey body sizes), the solid lines represent medium-sized prey (percentile 0.5 of prey body sizes), and dashed lines represent large prey (percentile 0.95 of prey body sizes). Selection coefficient relates prey consumption with prey traits. Selection of prey body sizes must be interpreted observing the vertical axis of the figure. When the dotted line is at the top of curves, small prey items are selected at this size class. When the dashed line is at the top, large size classes of prey are selected. Small prey items from all trophic groups are preferred by small predators. However, large predators select large primary producer prey over the small ones. In addition, the preference for small herbivorous-detritivores prey weakens for large predators, which consume this kind of prey independently of prey size. Note that large carnivorous prey are always avoided, and that this pattern is enhanced as predator body size increases.

The full CATS regression model outperformed each one of the alternative models (weight of evidence = 0.998) (S1 Table) showing a good performance (R^2^ = 0.67). This result indicates that 67% of the occurrence and strength of trophic links in this system was explained by the trait values of predators and prey here considered. Furthermore, our analysis sheds light on the prey selection mechanisms involved, by evidencing support for the three alternative mechanisms evaluated. In this context, our results support H_7_, which relates the combined action of the three prey selection mechanisms considered here. The action of the first mechanism about the increase in the number of prey consumed as a determinant of differences in trophic interactions along predator size classes was strongly supported. Specifically, the regression model with a random effect associated to predator body size class outperformed a model without this effect (ΔAIC = 505.33) (S1 Table). In addition, we obtained a strong positive association between the number of individual prey consumed and the predator body size (r = 0.91; t_18_ = 9.2; P < 0.0001). Despite this, we observed a change in the selection of prey traits (body size and trophic group) along the gradient of predator body size, also supporting the action of mechanisms M_2_ and M_3_ (Fig 3 and S2 Table and S4 Fig).

Supporting the prediction derived from the gape limitation mechanism, small predators positively selected small prey items from all trophic groups (i.e. β_small prey_ > β_large prey_) (Fig 3). However, this selection was progressively inverted for primary producer prey (β_small prey_ < β_large prey_) (Fig 3A) and reduced for herbivorous and detritivores prey (β_small prey_ ≈ β_large prey_) (Figs 3B and S4) as predator body size increased. Moreover, carnivorous prey of small size were always selected over large carnivorous prey (β_small prey_ > β_large prey_), a pattern that was magnified in large predators (Figs 3C and S4). In this sense, the gape limitation mechanism is supported by the consistent selection over small prey by small predators, and the selection pattern observed for herbivorous and detritivores prey in which both small and large prey items were equally consumed by large predators. Interestingly, the selection pattern observed for primary producers, for which large prey were preferred by large predators (Fig 3A), is the only one that supports the action of the optimal foraging mechanism in terms of selecting large over small prey by large predators. However, this mechanism is also supported by the observed replacement among prey trophic groups along size classes of predators (see below).

As expected under the optimal foraging mechanism, as predators become larger there was a transition in the selection pattern from low (i.e. primary producers) to high energy prey (i.e. animal prey−herbivorous, detritivores and carnivorous) (Fig 3). The selection for primary producers peaked at small size classes of predators (Fig 3A), whereas the selection for herbivorous and detritivores prey peaked at intermediate body size classes (Fig 3B). The reduction in the preference for herbivorous and detritivores prey was coupled with a progressive increase in the selection for carnivorous prey that continues rising towards the lager body size class of consumers. Consequently, selection of prey traits in terms of trophic group in the predator body size gradient goes from a preference for primary producer, which are replaced by the preference for herbivorous and detritivores prey, being finally replaced by carnivorous prey (Fig 3). In this sense, the consumption pattern supports the mechanism of optimal foraging towards more energetic prey as predators become large.

## Discussion

Prey selection is a basic process driving the emergence of food web structure [19, 56, 57]. Consequently, detecting the mechanisms that determine how and why prey are selected by predators is key to understand the structure and assembly of communities [2, 58]. In this framework, the increase in energy demand, the gape limitation and the optimal foraging mechanisms have been proposed to explain the role of consumers body size and prey traits on the emergence of common structural patterns among food webs. These mechanisms involve alternative trends in prey selection along predator body size gradients and were all supported by our results. Based on our analyses we found that: first, the energy demand mechanism, which refers to a trait-independent increase in number of prey at large body sizes, had strong statistical support as a determinant of trophic interactions. Second, selection on small prey for small size classes of predators associated with gape limitation is evidenced along primary producers, herbivorous and detritivores and carnivorous prey. This size preference is reduced for herbivorous and detritivores prey items as predator body size increases, additionally supporting the gape limitation mechanism. However, larger consumers progressively select smaller carnivorous prey, a pattern not expected from the hypotheses here and elsewhere evaluated and that requires considering further mechanisms—e.g. risk avoiding or enhanced escape behavior due to their higher mobility. Third, the replacement of selection among trophic categories in the gradient of consumer body size, and the switch in selection from small to large primary producer prey, support the action of the optimal foraging mechanism. From the set of seven hypotheses proposed, our results allowed to detect the most suitable one which combines the action of the three alternative mechanisms (H_7_). Our study shed light on the mechanisms through which the match-mismatch of predator-prey traits shapes food webs.

The taxonomic and functional diversity of the prey pool is the substrate over which selection mechanisms operate [35, 59]. A high prey diversity with a continuous representation of body sizes, as the observed in this system [14, 40]; S2 Fig), is optimal for evidencing different selection mechanisms. Congruently, clear trends in selection patterns were detected with a gradual transition in the sign and strength of trait selection from small to large predators. These patterns provide a direct evaluation of the main body size-dependent mechanisms proposed to explain food web architecture. It should be noted that the same selection pattern operating over a system with a low prey diversity and/or with discontinuous body sizes may have low effect on food web structure or promote sharp transition in consumption patterns [13, 27, 60]. In this vein, our study adds to the existing evidence, supporting that the pond food web herein analyzed is strongly structured by processes mediated by body size [12, 14, 40, 61, 62], and serves as an optimal study model for testing mechanisms based on trophic relationships guided by body size.

The CATS regression model exhibited a high explanatory power for the occurrence and strength of trophic interactions in the pond food web (R^2^ = 0.67). It is remarkable that only considering the body size of predators and prey, plus a general trophic categorization of these prey, was enough to explain a large fraction of the food web structure, in agreement with prior studies (e.g. [17, 63]). In addition, trait-selection analyses advance on the mechanistic understanding of the role of body size on the properties of food webs. For example, [14] detected a strong nested and antimodular pattern in this food web along the gradient in killifish body size. Nestedness has been also reported in several food webs [10, 11, 13, 64]. The gradual trend in selection of prey traits from smaller to larger consumers identifies the proximal mechanisms generating the observed nestedness in prey consumption [14]. However, it also points to a progressive substitution of prey trophic groups (Fig 3). Similarly, the relaxation in gape limitation and the increase in consumption rate explain the increase in trophic position, prey richness and number of energy sources with body size [12, 65]. It has to be highlighted that the trait selection approach is not intended to explain a particular food web pattern—e.g. nestedness, food chain length or connectance. However, 67% of the variation in weighted trophic links was explained by the model, explaining in a large extent the emergence of several features of food web architecture at higher levels. In this sense, the selection pattern of prey items should be central in modulating the stability of ecosystems, due to their connection with food webs features that were proved to determine network stability—modularity, nestedness, integration of energy channels [7, 12, 13, 17, 18, 63, 66].

The direct analysis of trait selection in trophic interactions was able to evidence the action of mechanisms not detected when the evaluation of particular food web metrics was the focus of different studies. For example, for the killifish guild herein studied, it was found that the preference of larger prey items was not a requirement for the emergence of body size-dependent food web structure [12]. Instead, the relaxation of gape limitation and the rise in prey number were able to explain the increase in prey diversity, energy paths and predator trophic position with body size. The selection pattern reported here further supports these ideas. However, trait selection analysis was also able to detect patterns congruent with optimal foraging predictions not revealed in previous studies (e.g. [12, 14]). In addition, a consistent selection for small sized carnivorous prey was here evidenced, being the consumption of large predatory prey always avoided. This trend is magnified for larger predators (Fig 3). Large carnivorous prey are less abundant [67], demand longer handling times [30], move faster and have more maneuverability [68, 69], and their consumption could be risky, either because of aggressive antipredator behavior [57, 70, 71] or due to trophically transmitted parasites [72]. The consistent avoidance of large carnivorous prey may be suggesting the action of mechanisms based on antipredator traits that would make them less profitable in terms of energy reward for large predators, in agreement with the optimal foraging mechanism [57]. In this sense, the use of CATS models for evidencing the mechanisms beyond the emergence of food web structure complements approaches based on specific metrics of food webs. Indeed, since the CATS regression predicts the elements of the weighted food web matrix, it could be potentially used for predicting a large variety of food web metrics commonly used in ecological studies.

Diet Weighted Mean (DWM) is a useful approach for evidencing how the representation of prey traits is changing along gradients of predators’ traits. The community weighted mean, from which the idea of DWM was borrowed, has shown a great potential for visualizing the action of possible assembly mechanisms [35, 51]. Although community weighted mean provides information regarding patterns, it is frequently used as the main tool for inferring the mechanisms behind them, representing an indirect way of testing hypotheses and their explanatory forces [73]. However, a single pattern of traits representation in a community can originate from different assembly rules [35]. Similarly, diet weighted means, prey frequencies or food web metrics can originate by several underlying causes. For example, considering that only a fraction of the total prey items (or traits) consumed could be truly subject to selection, the information provided by the general pattern of prey consumption shown in Fig 2 is insufficient to tell between those selected and not selected prey items and the potential explanations for such selection [74]. The trait-dependent prey selection approach overcomes these limitations, providing a direct analysis of the selection processes that determine trends in diet among predators or environments. Finally, traits selection models can also overcome a canonical limitation of prey preference studies about the need to identify the species pool truly available for consumers to evidence selection mechanisms [75–77]. In the CATS regression model, the set of prey items and abundances observed among all consumers can be used as the effective species pool exploited by predators. This effective prey species pool is included as both an offset and as a random factor that indicates the expected prey frequencies in each predator body size class. Therefore, changes in the representation of these items in the diet of predators with different body sizes can be associated with a selection process based on the interaction between consumers and prey traits [36]. The CATS model represents a robust tool to analyze prey selection mechanisms based on functional, morphological and behavioral attributes that complements previous approach and overcomes some limitations from behavioral ecology and food web studies. This is key in the inference process and hypothesis testing contributing to the progress in the field of food webs [78–80].

## Conclusions

Our study focused on the mechanistic basis responsible for several well-reported trends in food web structure with predator body size. These trends can be explained by different mechanisms involving trait-mediated interactions between predators and their prey, which have been indirectly inferred in the past. By using a trait-based approach we found support for the three proposed mechanisms associated with energy demand, gape limitation and optimal foraging, but also for additional mechanisms such as risk avoidance as determinants of food web structure. On this basis, our results favored the most complex of the tested hypotheses, H7, which integrates the three prey selection mechanisms considered in this study. Unveiling the connection between species traits and trophic interactions has the potential to provide mechanistic predictions about the expected effect of changes in species traits, driven either by changes in community composition or by trait evolution, on the structure of food webs.

## Supporting information

S1 Table Model selection process results. Akaike´s Information Criterion values (AIC), weight of evidence (WAIC) and Wilks´ likelihood ratio test results for the alternative models included in the selection process. Predator body size (PredBS), Predator body size^2 (PredBS2), Prey body size (PreyBS), Prey trophic group (PreyTG), Predator body size class identity (PredBSC ID).
(XLSX)

S2 Table CATS regression model results. Selection coefficients obtained from the CATS model (R^2^ = 0.67). Body size (BS), Trophic group (TG).
(XLSX)

S1 Fig Predator body size classes and their range values. Range values for the 20 predator body size classes used in the analyses. Each body size class is composed of 31 killifish individuals, except for the last one which contains 30 individuals.
(TIF)

S2 Fig Prey body size distribution. Body size distribution of potential animal prey categorized as A) herbivorous-detritivores and B) carnivorous in the study system. Body size estimations were made from a dataset of invertebrate and vertebrate individuals collected in 2008 in the study system (see Methods section). The green line represents the body size range of primary producer prey, which was obtained from published literature.
(TIF)

S3 Fig Prey richness and abundance. Richness (A) and abundance (B) of prey items categorized as primary producers, herbivorous-detritivores and carnivorous in the diet of each body size class of killifish.
(TIF)

S4 Fig Selection of prey body size along the predator body size gradient. Selection of prey body size along predator body size classes for prey categorized as (A) primary producers, (B) herbivorous and detritivores and (C) carnivorous. Selection coefficient relates prey consumption based on their size with predator body size. Small predators select over small prey items of all trophic groups. However, large predators select larger primary producers meanwhile they consume herbivorous-detritivores prey items independently of their size. Contrary, as predator body size increases, large carnivorous prey are always avoided, a pattern that is magnified for those larger predators.
(TIF)

## References

[1] Brose U, Archambault P, Barnes AD, Bersier L-F, Boy T, Canning-Clode J, et al. Predator traits determine food-web architecture across ecosystems. Nat Ecol Evol. 2019;3: 919–927. doi: 10.1038/s41559-019-0899-x 31110252

[2] Hogle SL, Hepolehto I, Ruokolainen L, Cairns J, Hiltunen T. Effects of phenotypic variation on consumer coexistence and prey community structure. Ecology Letters. 2022;25: 307–319. doi: 10.1111/ele.13924 34808704 PMC9299012

[3] Miller-ter Kuile A, Apigo A, Bui A, DiFiore B, Forbes ES, Lee M, et al. Predator–prey interactions of terrestrial invertebrates are determined by predator body size and species identity. Ecology. 2022;103: e3634. doi: 10.1002/ecy.3634 35060625

[4] Berlow EL, Dunne JA, Martinez ND, Stark PB, Williams RJ, Brose U. Simple prediction of interaction strengths in complex food webs. PNAS. 2009;106: 187–191. doi: 10.1073/pnas.0806823106 19114659 PMC2629248

[5] Boukal DS. Trait- and size-based descriptions of trophic links in freshwater food webs: current status and perspectives. Journal of Limnology. 2014;73. doi: 10.4081/jlimnol.2014.826

[6] Brose U, Blanchard JL, Eklöf A, Galiana N, Hartvig M, Hirt M R., et al. Predicting the consequences of species loss using size-structured biodiversity approaches. Biological Reviews. 2017;92: 684–697. doi: 10.1111/brv.12250 26756137

[7] Brose U, Williams RJ, Martinez ND. Allometric scaling enhances stability in complex food webs. Ecology Letters. 2006;9: 1228–1236. doi: 10.1111/j.1461-0248.2006.00978.x 17040325

[8] Cohen JE, Pimm SL, Yodzis P, Saldaña J. Body Sizes of Animal Predators and Animal Prey in Food Webs. Journal of Animal Ecology. 1993;62: 67–78. doi: 10.2307/5483

[9] Cohen JE, Jonsson T, Carpenter SR. Ecological community description using the food web, species abundance, and body size. Proceedings of the National Academy of Sciences. 2003;100: 1781–1786. doi: 10.1073/pnas.232715699 12547915 PMC149910

[10] Woodward G, Hildrew AG. Body-size determinants of niche overlap and intraguild predation within a complex food web. Journal of Animal Ecology. 2002;71: 1063–1074. doi: 10.1046/j.1365-2656.2002.00669.x

[11] Araújo MS, Martins EG, Cruz LD, Fernandes FR, Linhares AX, Dos Reis SF, et al. Nested diets: a novel pattern of individual-level resource use. Oikos. 2010;119: 81–88. doi: 10.1111/j.1600-0706.2009.17624.x

[12] Arim M, Abades SR, Laufer G, Loureiro M, Marquet PA. Food web structure and body size: trophic position and resource acquisition. Oikos. 2010;119: 147–153. doi: 10.1111/j.1600-0706.2009.17768.x

[13] Ramos-Jiliberto R, Valdovinos FS, Arias J, Alcaraz C, García-Berthou E. A network-based approach to the analysis of ontogenetic diet shifts: An example with an endangered, small-sized fish. Ecological Complexity. 2011;8: 123–129. doi: 10.1016/j.ecocom.2010.11.005

[14] Ortiz E, Arim M. Hypotheses and trends on how body size affects trophic interactions in a guild of South American killifishes: Trophic Insertion and Body Size. Austral Ecology. 2016;41: 976–982. doi: 10.1111/aec.12389

[15] Nordström MC, Aarnio K, Törnroos A, Bonsdorff E. Nestedness of trophic links and biological traits in a marine food web. Ecosphere. 2015;6: art161. doi: 10.1890/ES14-00515.1

[16] Williams RJ, Martínez ND. Simple rules yield complex food webs. Nature. 2000;404: 180–183. doi: 10.1038/35004572 10724169

[17] Otto SB, Rall BC, Brose U. Allometric degree distributions facilitate food-web stability. Nature. 2007;450: 1226–1229. doi: 10.1038/nature06359 18097408

[18] Petchey OL, Beckerman AP, Riede JO, Warren PH. Size, foraging, and food web structure. Proceedings of the National Academy of Sciences. 2008;105: 4191–4196. doi: 10.1073/pnas.0710672105 18337512 PMC2393804

[19] Stouffer DB, Camacho J, Jiang W, Nunes Amaral LA. Evidence for the existence of a robust pattern of prey selection in food webs. Proceedings of the Royal Society B: Biological Sciences. 2007;274: 1931–1940. doi: 10.1098/rspb.2007.0571 17567558 PMC2275185

[20] Arim M, Bozinovic F, Marquet PA. On the relationship between trophic position, body mass and temperature: reformulating the energy limitation hypothesis. Oikos. 2007;116: 1524–1530. doi: 10.1111/j.0030-1299.2007.15768.x

[21] McNab BK. The Physiological Ecology of Vertebrates: A View from Energetics. Cornell University Press; 2002.

[22] Brown JH, Gillooly JF, Allen AP, Savage VM, West GB. Toward a metabolic theory of ecology. Ecology. 2004;85: 1771–1789. doi: 10.1890/03-9000

[23] Hairston NG. Cause-Effect Relationships in Energy Flow, Trophic Structure, and Interspecific Interactions. The American Naturalist. 1993;142: 379–411.

[24] Layman CA, Winemiller KO, Arrington DA, Jepsen DB. Body Size and Trophic Position in a Diverse Tropical Food Web. Ecology. 2005;86: 2530–2535. doi: 10.1890/04-1098

[25] Mittelbach GG. Foraging Efficiency and Body Size: A Study of Optimal Diet and Habitat Use by Bluegills. Ecology. 1981;62: 1370–1386. doi: 10.2307/1937300

[26] Carbone C, Mace GM, Roberts SC, Macdonald DW. Energetic constraints on the diet of terrestrial carnivores. Nature. 1999;402: 286–288. doi: 10.1038/46266 10580498

[27] Sherwood GD, Kovecses J, Hontela A, Rasmussen JB. Simplified food webs lead to energetic bottlenecks in polluted lakes. Can J Fish Aquat Sci. 2002;59: 1–5. doi: 10.1139/f01-213

[28] Brose U, Cushing L, Berlow EL, Jonsson T, Banasek-Richter C, Bersier L-F, et al. Body Sizes of Consumers and Their Resources. Ecology. 2006;86: 2545–2545. doi: 10.1890/05-0379

[29] Sih A. Optimal Foraging: Partial Consumption of Prey. The American Naturalist. 1980;116: 281–290. doi: 10.1086/283626

[30] Lazzaro X. A review of planktivorous fishes: Their evolution, feeding behaviours, selectivities, and impacts. Hydrobiologia. 1987;146: 97–167. doi: 10.1007/BF00008764

[31] Werner EE, Hall DJ. Optimal Foraging and the Size Selection of Prey by the Bluegill Sunfish (Lepomis Macrochirus). Ecology. 1974;55: 1042–1052. doi: 10.2307/1940354

[32] Schael DM, Rudstam LG, Post JR. Gape Limitation and Prey Selection in Larval Yellow Perch (Perca flavescens), Freshwater Drum (Aplodinotus grunniens), and Black Crappie (Pomoxis nigromaculatus). Can J Fish Aquat Sci. 1991;48: 1919–1925. doi: 10.1139/f91-228

[33] Devries DR, Stein RA, Bremigan MT. Prey Selection by Larval Fishes as Influenced by Available Zooplankton and Gape Limitation. Transactions of the American Fisheries Society. 1998;127: 1040–1050. doi: 10.1577/1548-8659(1998)127<1040:PSBLFA>2.0.CO;2

[34] Shipley B, Vile D, Garnier É. From Plant Traits to Plant Communities: A Statistical Mechanistic Approach to Biodiversity. Science. 2006;314: 812–814. doi: 10.1126/science.1131344 17023613

[35] Shipley B. Community assembly, natural selection and maximum entropy models. Oikos. 2010;119: 604–609. doi: 10.1111/j.1600-0706.2009.17770.x

[36] Warton DI, Shipley B, Hastie T. CATS regression–a model-based approach to studying trait-based community assembly. Methods in Ecology and Evolution. 2015;6: 389–398. doi: 10.1111/2041-210X.12280

[37] Cunillera-Montcusí D, Arim M, Gascón S, Tornero I, Sala J, Boix D, et al. Addressing trait selection patterns in temporary ponds in response to wildfire disturbance and seasonal succession. Journal of Animal Ecology. 2020;89: 2134–2144. doi: 10.1111/1365-2656.13265 32441323

[38] Griffiths D. Prey Availability and the Food of Predators. Ecology. 1975;56: 1209–1214. doi: 10.2307/1936161

[39] Hart P, Hamrin SF. Pike as a Selective Predator. Effects of Prey Size, Availability, Cover and Pike Jaw Dimensions. Oikos. 1988;51: 220–226. doi: 10.2307/3565645

[40] Arim M, Berazategui M, Barreneche JM, Ziegler L, Zarucki M, Abades SR. Determinants of Density–Body Size Scaling Within Food Webs and Tools for Their Detection. In: Belgrano A, editor. Advances in Ecological Research. Academic Press; 2011. pp. 1–39. doi: 10.1016/B978-0-12-386475-8.00001-0

[41] Keppeler FW, Lanés LEK, Rolon AS, Stenert C, Lehmann P, Reichard M, et al. The morphology–diet relationship and its role in the coexistence of two species of annual fishes. Ecology of Freshwater Fish. 2015;24: 77–90. doi: 10.1111/eff.12127

[42] Laufer G, Arim M, Loureiro M, Piñeiro-Guerra JM, Clavijo-Baquet S, Fagúndez C. Diet of four annual killifishes: an intra and interspecific comparison. Neotropical Ichthyology. 2009;7: 77–86. doi: 10.1590/S1679-62252009000100010

[43] Illarze M, Grandal EO, Rodriguez-Tricot L, Pinelli V, Piñeiro-Guerra JM, Sosa-Panzera L, et al. La diversidad escondida: invertebrados de charcos temporales en Barra Grande, Uruguay. Boletín de la Sociedad Zoológica del Uruguay. 2021;30: e30.2.5-e30.2.5. doi: 10.26462/30.2.5

[44] Ziegler L, Berazategui M, Arim M. Discontinuities and alternative scalings in the density–mass relationship of anuran larvae. Hydrobiologia. 2014;723: 123–129. doi: 10.1007/s10750-013-1553-2

[45] Lombardo A. Flora montevidensis ‐ Tomo I. Montevideo: IMM; 1982.

[46] Lombardo A. Flora montevidensis ‐ Tomo II ‐ Gamopétalas. Montevideo: IMM; 1983.

[47] Lombardo A. Flora montevidensis ‐ Tomo III ‐ Monocotiledóneas. Montevideo: IMM; 1984.

[48] Alonso Paz E. Plantas acuáticas de los humedales del este. Montevideo: Probides; 1997.

[49] Kruk C, Huszar VLM, Peeters ETHM, Bonilla S, Costa L, Lürling M, et al. A morphological classification capturing functional variation in phytoplankton. Freshwater Biology. 2010;55: 614–627. doi: 10.1111/j.1365-2427.2009.02298.x

[50] Lavorel S, Grigulis K, McIntyre S, Williams NSG, Garden D, Dorrough J, et al. Assessing functional diversity in the field–methodology matters! Functional Ecology. 2008;22: 134–147. doi: 10.1111/j.1365-2435.2007.01339.x

[51] ter Braak CJF. New robust weighted averaging- and model-based methods for assessing trait–environment relationships. Methods in Ecology and Evolution. 2019;10: 1962–1971. doi: 10.1111/2041-210X.13278

[52] Braga J, ter Braak CJF, Thuiller W, Dray S. Integrating spatial and phylogenetic information in the fourth-corner analysis to test trait–environment relationships. Ecology. 2018;99: 2667–2674. doi: 10.1002/ecy.2530 30289571

[53] Brown AM, Warton DI, Andrew NR, Binns M, Cassis G, Gibb H. The fourth-corner solution–using predictive models to understand how species traits interact with the environment. Methods in Ecology and Evolution. 2014;5: 344–352. doi: 10.1111/2041-210X.12163

[54] Burnham KP, Anderson DR. Model Selection and Multimodel Inference: A Practical Information-Theoretic Approach. 2nd ed. New York: Springer-Verlag; 2002. Available: //www.springer.com/la/book/9780387953649

[55] R Development Core Team. R: A language and environment for statistical computing. Vienna, Austria.: R Foundation for Statistical Computing, Vienna, Austria. ISBN 3-900051-07-0, URL http://www.R-project.org.; 2021. Available: http://www.R-project.org.

[56] Green SJ, Côté IM. Trait-based diet selection: prey behaviour and morphology predict vulnerability to predation in reef fish communities. Journal of Animal Ecology. 2014;83: 1451–1460. doi: 10.1111/1365-2656.12250 24861366

[57] Predator Schmitz O. and prey functional traits: understanding the adaptive machinery driving predator–prey interactions. F1000Res. 2017;6: 1767. doi: 10.12688/f1000research.11813.1 29043073 PMC5621104

[58] Rossberg AG, Brännström Å, Dieckmann U. How trophic interaction strength depends on traits. Theor Ecol. 2010;3: 13–24. doi: 10.1007/s12080-009-0049-1

[59] Vellend M. The Theory of Ecological Communities. Princeton University Press; 2016. Available: https://press.princeton.edu/titles/10914.html

[60] Lewinsohn TM, Prado PI, Jordano P, Bascompte J, Olesen JM. Structure in plant–animal interaction assemblages. Oikos. 2006;113: 174–184. doi: 10.1111/j.0030-1299.2006.14583.x

[61] Borthagaray AI, Berazategui M, Arim M. Disentangling the effects of local and regional processes on biodiversity patterns through taxon‐contingent metacommunity network analysis. Oikos. 2015;124: 1383–1390. doi: 10.1111/oik.01317

[62] Canavero A, Hernández D, Zarucki M, Arim M. Patterns of co-occurrences in a killifish metacommunity are more related with body size than with species identity. Austral Ecology. 2014;39: 455–461. doi: 10.1111/aec.12103

[63] Beckerman AP, Petchey OL, Warren PH. Foraging biology predicts food web complexity. Proceedings of the National Academy of Sciences. 2006;103: 13745–13749. doi: 10.1073/pnas.0603039103 16954193 PMC1560085

[64] Pires MM, Guimarães PR Jr, Araújo MS, Giaretta AA, Costa JCL, dos Reis SF. The nested assembly of individual-resource networks. Journal of Animal Ecology. 2011;80: 896–903. doi: 10.1111/j.1365-2656.2011.01818.x 21644976

[65] Rooney N, McCann KS. Integrating food web diversity, structure and stability. Trends in Ecology & Evolution. 2012;27: 40–46. doi: 10.1016/j.tree.2011.09.001 21944861

[66] Abrams PA. Implications of flexible foraging for interspecific interactions: lessons from simple models. Functional Ecology. 2010;24: 7–17. doi: 10.1111/j.1365-2435.2009.01621.x

[67] Damuth J. Population density and body size in mammals. Nature. 1981;290: 699–700. doi: 10.1038/290699a0

[68] Hirt MR, Lauermann T, Brose U, Noldus LPJJ, Dell AI. The little things that run: a general scaling of invertebrate exploratory speed with body mass. Ecology. 2017;98: 2751–2757. doi: 10.1002/ecy.2006 28887816

[69] Martin BT, Gil MA, Fahimipour AK, Hein AM. Informational constraints on predator–prey interactions. Oikos. 2022;2022: e08143. doi: 10.1111/oik.08143

[70] Mukherjee S, Heithaus MR. Dangerous prey and daring predators: a review. Biological Reviews. 2013;88: 550–563. doi: 10.1111/brv.12014 23331494

[71] Ho H-C, Tylianakis JM, Zheng JX, Pawar S. Predation risk influences food-web structure by constraining species diet choice. Ecology Letters. 2019;22: 1734–1745. doi: 10.1111/ele.13334 31389145

[72] Lafferty KD, Dobson AP, Kuris AM. Parasites dominate food web links. Proceedings of the National Academy of Sciences. 2006;103: 11211–11216. doi: 10.1073/pnas.0604755103 16844774 PMC1544067

[73] Spasojevic MJ, Suding KN. Inferring community assembly mechanisms from functional diversity patterns: the importance of multiple assembly processes. Journal of Ecology. 2012;100: 652–661. doi: 10.1111/j.1365-2745.2011.01945.x

[74] Mascaro M, Hidalgo LE, Chiappa-Carrara X, Simoes N. Size-selective foraging behaviour of blue crabs, Callinectes sapidus (Rathbun), when feeding on mobile prey: Active and passive components of predation. Marine and Freshwater Behaviour and Physiology. 2003;36: 143–159. doi: 10.1080/10236240310001603224

[75] Sánchez-Hernández J, Nunn AD, Adams CE, Amundsen P-A. Causes and consequences of ontogenetic dietary shifts: a global synthesis using fish models. Biological Reviews. 2019;94: 539–554. doi: 10.1111/brv.12468 30251433

[76] Fairweather PG. Experiments on the interaction between predation and the availability of different prey on rocky seashores. Journal of Experimental Marine Biology and Ecology. 1988;114: 261–273. doi: 10.1016/0022-0981(88)90142-6

[77] Ceron K, Provete DB, Pires MM, Araujo AC, Blüthgen N, Santana DJ. Differences in prey availability across space and time lead to interaction rewiring and reshape a predator–prey metaweb. Ecology. 2022;103: e3716. doi: 10.1002/ecy.3716 35388458

[78] Platt JR. Strong Inference. Science. 1964;146: 347–353. doi: 10.1126/science.146.3642.347 17739513

[79] Marquet PA, Allen AP, Brown JH, Dunne JA, Enquist BJ, Gillooly JF, et al. On Theory in Ecology. BioScience. 2014;64: 701–710. doi: 10.1093/biosci/biu098

[80] Betts MG, Hadley AS, Frey DW, Frey SJK, Gannon D, Harris SH, et al. When are hypotheses useful in ecology and evolution? Ecology and Evolution. 2021;11: 5762–5776. doi: 10.1002/ece3.7365 34141181 PMC8207363

