# Correction: Prey selection along a predators’ body size gradient evidences the role of different trait-based mechanisms in food web organization

**DOI:** 10.1371/journal.pone.0317143

**Published:** 2025-01-03

**Authors:** Esteban Ortiz, Rodrigo Ramos-Jiliberto, Matías Arim

The Funding statement for this paper is incorrect. The correct Funding statement is as follows: This research was funded by Horizon 2020 Framework Programme (Grant Number 869296) (https://cordis.europa.eu/project/id/869296). Additional financial support was provided by: Agencia Nacional de Investigación e Innovación (https://www.anii.org.uy/) (Fondo Clemente Estable, grant number 2014–104763) and CSIC-grupos (ID 657725) to MA; Comisión Académica de Posgrado (https://www.posgrados.udelar.edu.uy/cap.php), grant numbers BDDX_2019_1#49295789 and BFPD_2022_1#49295789 and CSIC_Iniciación_2019_ID_36 to EO; Agencia Nacional de Investigación y Desarrollo/Fondo Nacional de Desarrollo Científico y Tecnológico (https://www.anid.cl/), grant number 1231321 to RRJ. The founders had no role in study design, data collection and analysis, decision to publish, or preparation of the manuscript.

## References

[1] Ortiz E, Ramos-Jiliberto R, Arim M (2023) Prey selection along a predators’ body size gradient evidences the role of different trait-based mechanisms in food web organization. PLOS ONE 18(10): e0292374. 10.1371/journal.pone.0292374 37797081 PMC10553361

