# Supplementary figures and images for: Prey selection along a predators’ body size gradient evidences the role of different trait-based mechanisms in food web organization
Source: PLoS One. 2023 Oct 5;18(10):e0292374. doi: 10.1371/journal.pone.0292374 (PMC10553361; doi:10.1371/journal.pone.0292374)

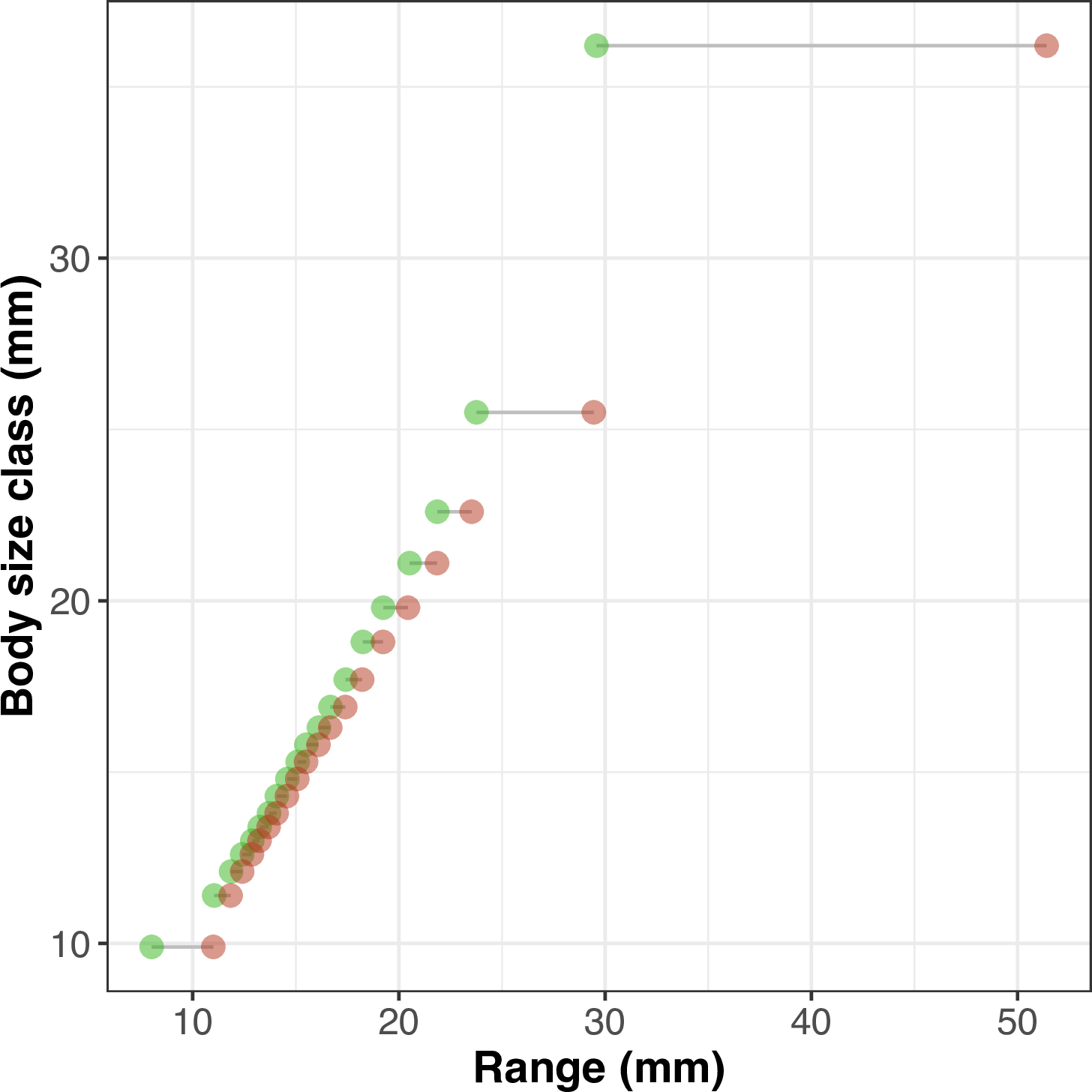

Supplement: S1 Fig — Range values for the 20 predator body size classes used in the analyses. Each body size class is composed of 31 killifish individuals, except for the last one which contains 30 individuals. (TIF) [file pone.0292374.s003.tif]

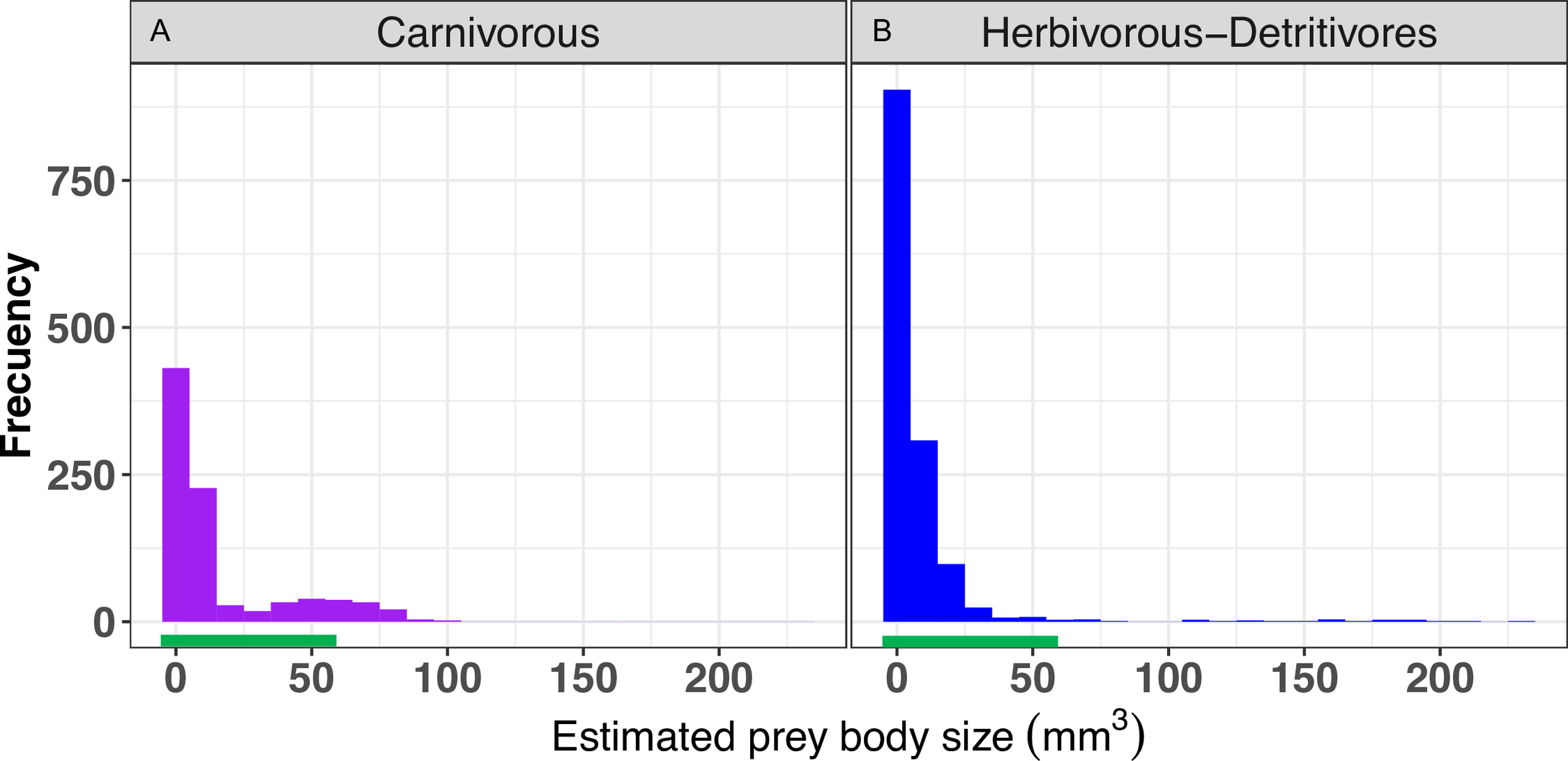

Supplement: S2 Fig — Body size distribution of potential animal prey categorized as A) herbivorous-detritivores and B) carnivorous in the study system. Body size estimations were made from a dataset of invertebrate and vertebrate individuals collected in 2008 in the study system (see Methods section). The green line represents the body size range of primary producer prey, which was obtained from published literature. (TIF) [file pone.0292374.s004.tif]

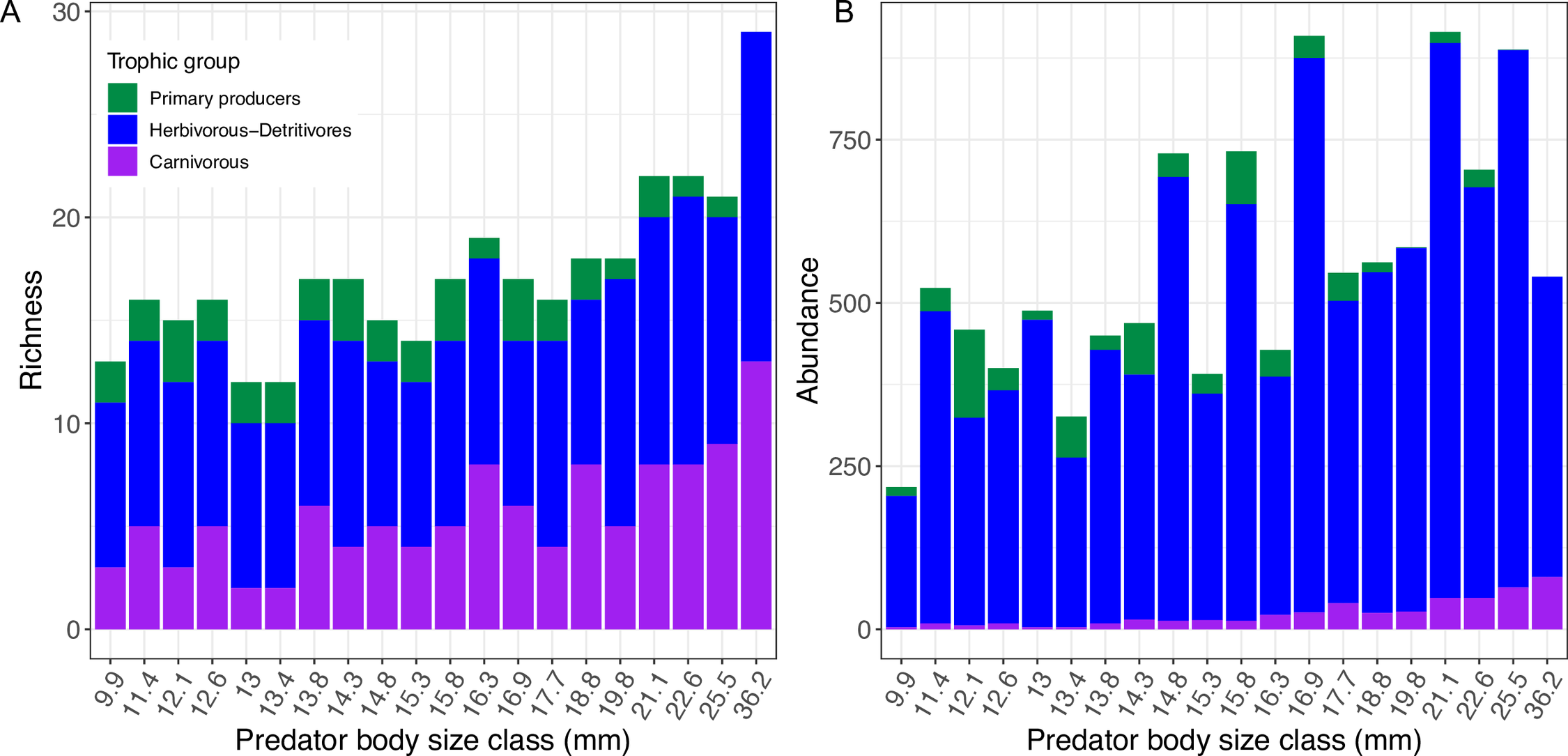

Supplement: S3 Fig — Richness (A) and abundance (B) of prey items categorized as primary producers, herbivorous-detritivores and carnivorous in the diet of each body size class of killifish. (TIF) [file pone.0292374.s005.tif]

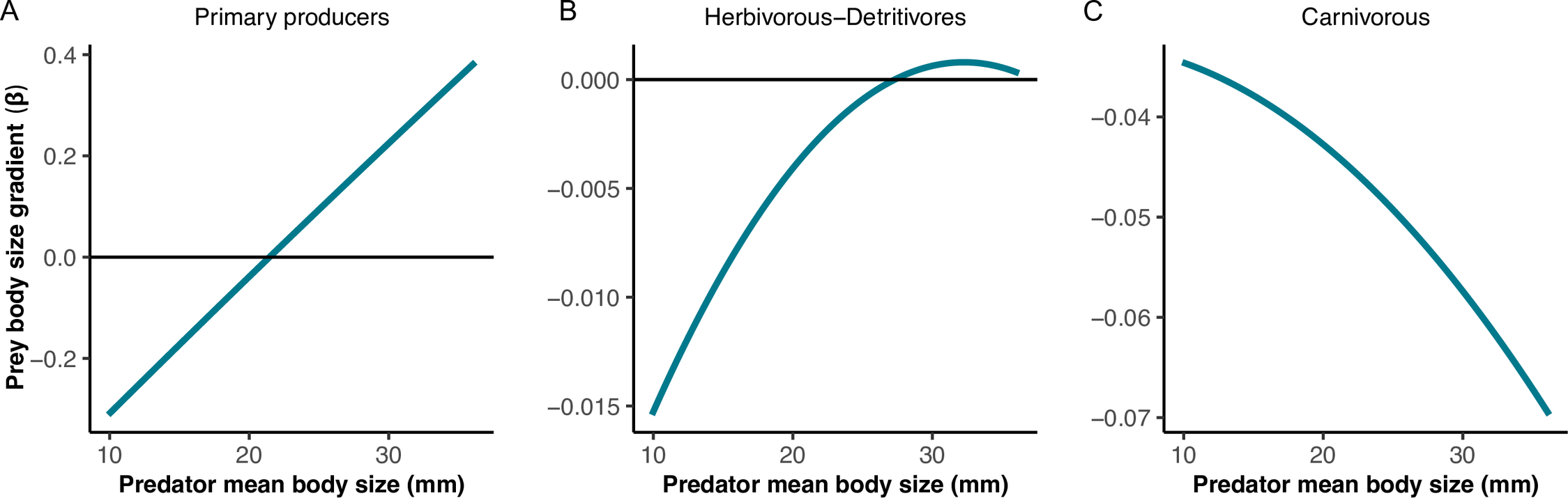

Supplement: S4 Fig — Selection of prey body size along predator body size classes for prey categorized as (A) primary producers, (B) herbivorous and detritivores and (C) carnivorous. Selection coefficient relates prey consumption based on their size with predator body size. Small predators select over small prey items of all trophic groups. However, large predators select larger primary producers meanwhile they consume herbivorous-detritivores prey items independently of their size. Contrary, as predator body size increases, large carnivorous prey are always avoided, a pattern that is magnified for those larger predators. (TIF) [file pone.0292374.s006.tif]
